# The multiple tenses of a postcolonial age of migration: a commentary on Samaddar, R. (2020).The postcolonial age of migration London and New York: Taylor & Francis, 2020

**DOI:** 10.1007/s10624-021-09619-4

**Published:** 2021-03-14

**Authors:** Ayşe Çağlar

**Affiliations:** University of Vienna/Institute for Human Sciences, Vienna, Austria

“Imperial effects occupy multiple historical tenses. They are at once product of the past imperfect that selectively permeate the present and they shape both the conditional subjunctive and uncertain futures” (Ann Laura Stoler, *Imperial Debris: On Ruins and Ruination*, 2013, p. 10)

*The Postcolonial Age of Migration* is a clarion call to unravel the colonial gradient in today’s population flows and migration regimes. Showing the tangibility of colonial pasts and imperial presences in today’s mechanisms and strategies of population control and politics, the book poses a provocative challenge to studies that fail to see the colonial/imperial debris in the making and remaking of today’s migrations and their governance. The realities of partition, the colonial state, decolonization, the departure of colonial power, and border drawings and their long-term effects take center stage in Samaddar’s thoughtful and provocative intervention. He argues that we need a postcolonial perspective to extricate their visceral presence and effects in the age of migration, which in turn requires the acknowledgement of imperial lineages in nations’ histories. Partition and its shadow lines are at the heart of forced displacements.

*The Postcolonial Age of Migration* takes off from a disappointment with Stephen Castles and Mark J. Millers’ (2003 and 2009) seminal work, *The Age of Migration*. Despite its global perspective, according to Samaddar, that work fails to grasp the specificity of the age of migration as it misses the impact of colonial formations in the making of the population movements of this age. It was in the colonial age that the governing principles of mobility and the control and governance of mobile bodies, labor, population, and cities were laid down. That is why what Castles and Mills identify as the age of migration is, in fact, a postcolonial one. The flexible frontier policies and the governance mechanisms of the colonial age, which not only functioned to regulate migration but also produced population flows, clearly shaped nation states. “The postcolonial impact has been mainly in the form of combining a nation state’s border and security strategy with old imperial-colonial strategy of maintaining expansive security lines and zones, keeping virtual borderlands, expanses of indeterminate zones, and as result of these a flexible frontier policy” (p. 154).

Samaddar’s critique directed at *The Age of Migration* is particularly important because Castles and Millers’ book has been central for the coming-of-age of critical migration scholars, particularly those in the global North who desired to move migration scholarship and public debates beyond the migration-return-integration-social cohesion stalemate in mainstream studies. *The Postcolonial Age of Migration* seeks to animate an analytical vocabulary beyond the established repertoires of traditional and critical migration studies. This forms the backbone of Samaddar’s critical reading of the contemporary migration dynamics and regimes in relation to various colonial practices, tools of population control, and governance mechanisms. It is this vocabulary that enables him to chronicle and analyze the relations and connections between the dynamics of migration, colonialism, states, empires, power, border and frontier formations, and various forms of forced migration together.

My reading of Samaddar’s work evoked Amitav Ghosh’s acclaimed novel, *The Shadow Lines* (2010, [1988]), in which we are moved to think about the afterlife of forced displacements in the lives of people. The elusive legacies of partition have a tenacious hold on people’s lives and memories, and Gosh skillfully shows this through the entangled histories and stories of two families. *The Shadow Lines* collapses time and space, placing events from different times and places next to each other as if it were impossible to tell these stories outside of their entanglements and without shifting between multiple tenses. As Ranabir Samaddar’s *The Postcolonial Age of Migration* shows, this is indeed impossible and so every page from the first to the last pages of Samaddar’s book, *The Shadow Lines* for me is its silent barely visible companion. In contrast to the elusive presence of *Shadow Lines*, Raymond Williams’ (2014 [1976]) influential *Keywords* is a very visible companion of Samaddar’s endeavor. However, the keywords in *The Postcolonial Age of Migration* neither elaborate the time-space-bound variations of the central terms of migration scholarship and governance nor aim at a geographical extension to places with a colonial past. Instead of detailing selected keywords and their variations, Samaddar maps out families of keywords to track and analyze the relations between the entangled dynamics and processes of migration, colonialism, states, and border and frontier formations. Indeed, it is these different clusters that set the intellectual and public worlds of today’s scholarship and public debates on migrants and migration regimes apart.

Samaddar’s analytical vocabulary is less about differentiating categories of forced migration and tracing their genealogies through a rigid grid to sort out refugees, internally displaced people, and environmental and economic migrants that is prominent in the circles of international organizations, governments, policy, and non-government organizations and much scholarship. Rather, it is more about the futility of such efforts. It is a call to recognize the massive and mixed nature of population movements. As such, *The Postcolonial Age of Migration* is more than a book about decentering the histories and genealogies of the concept of forced migration.

The challenge of disentangling the postcolonial gradient of today’s world of migrations lies not in highlighting the colonial leftovers and imprints or a generic indictment of colonial history, but in explicating the active and ongoing force of colonial/imperial formations in contemporary processes of population movements and their governance. As Ann Laura Stoler elegantly put it in her 2013 book, imperial debris could only be thought in multiple tenses but never as a completed *passé composé*. A failure to note and analyze the reappropriation and the active lives of the imperial/colonial formations in the politics of the present easily misses the multiple historical tenses of these processes.

The governance technologies of imperial rule were strongly anchored in differentiated and racialized access and rights in colonial frontiers. Samaddar writes: “For population to be managed and population groups to be stabilized and governed, colonial rule needed flexible border policies. Such policy allowed expansion. It included administrative policies for footloose population groups, and more importantly, measures that enabled shifting of border in case of need” (p. 150). The gradated forms of sovereignty of the colonial frontier policies that ensured the flexible management of labor supply while establishing the regulatory principle of colonial rule were taken over by modern nation states. Today’s control and governance of labor mobility that produce migrant labor as politically invisible group without rights were laid down in colonial and imperial rule.

*The Postcolonial Age of Migration* was written long before the Covid-19 pandemic.^1^ However, its insights in the time of the Covid-19 pandemic are evident as we see the postcolonial gradient in today’s labor and population movements and their governance. Little observational skill is needed to detect the increasing reign of flexible frontier strategies and mechanisms in today’s migration and border governance. The closures of European borders and the variegated permeability within and of these vis-à-vis labor mobility in the wake of Covid-19, as well as the fractured legal geographies and administrative maze of refugee camps on Greek islands, show how well and alive these frontier policies are. Every strict closure at the height of the pandemic ensured that the borders remained differentially open to seasonal and temporary (predominantly agricultural, domestic, and health care) workers with an armature of exceptional administrative and surveillance systems and techniques put in place.

The vivid presence of these dynamics casts no doubt on Samaddar’s contention that that our age is a postcolonial age of migration. However, the challenge remains in terms of tracking and analyzing the ways these formations work their way not only through contemporary inequalities and into politics but also into forms of resistance and alliances. If we see the resurrection of these structures and relations in the contemporary governance and its strategies of control over populations, it might also be important to scrutinize their inroads into community-based forms of care in ways that problematize the entanglements of such forms of care with colonial formations. This is important to pursue in connection with  a politics that offers alternatives. A group of migration scholars are now picking up this challenge in light of the orientation *The Postcolonial Age of Migration* provides.^2^ Rather than simply adding the histories and dynamics of forced migrations in Asia to the almost canonized questions of migration scholarship and debates in Europe or vice versa, many such scholars are seeking to shift the analytical lens to ask different questions about forced migration, migrant labor, and their contradictions and entanglements, as well as borders and, above all, politics in Europe and Asia. As Samaddar argues, a postcolonial perspective is the prerequisite for a truly global understanding of migration.

## Footnotes

1 Parts of it were written during Ranabir Samaddar’s fellowships at the Institute for Human Sciences (IWM). It was made in IWM (https://www.iwm.at/always-active/book-by-fellows/made-in-iwm-ranabir-samaddar-the-postcolonial-age-of-migration/).

2 See for example The Europe-Asia Research platform on Forced Migration at IWM and the Mahanirban Calcutta Research Group (https://www.iwm.at/research/projects/europe-asia-research-platform-on-forced-migration/).
